# Outbreak of Serogroup W135 Meningococcal Disease after the Hajj Pilgrimage, Europe, 2000

**DOI:** 10.3201/eid0808.010422

**Published:** 2002-08

**Authors:** Jean-François Aguilera, Anne Perrocheau, Christine Meffre, Susan Hahné, the W135 Working Group

**Affiliations:** * Communicable Disease Surveillance Centre, London, United Kingdom; † Institut de Veille Sanitaire, St. Maurice, France; ‡ Rijksinstituut voor Volksgezondheid en Milieu (RIVM), European Programme for Intervention Epidemiology Training, Bilthoven, the Netherlands; § Communicable Disease Surveillance Centre, Cardiff, Wales, United Kingdom

**Keywords:** *Neisseria meningitidis* W135, outbreaks, the Hajj, mortality, chemoprophylaxis

## Abstract

The 2000 Hajj (March 15–18) was followed by an outbreak of *Neisseria meningitidis* W135 2a: P1.2,5 in Europe. From March 18 to July 31, 2000, some 90 cases of meningococcal infection were reported from nine countries, mostly the United Kingdom (UK) and France; 14 cases were fatal. Although most early cases were in pilgrims, the outbreak spread to their contacts and then to those with no known pilgrim contact. In France and the UK, the outbreak case-fatality rate was compared with the rate reported from national surveillance. The risk of dying during this outbreak was higher in France and the UK, although the difference was not statistically significant. Prophylaxis for all pilgrims and their household contacts was offered in France; in the UK and other European countries, prophylaxis was recommended only for close contacts. No difference in transmission rates following intervention was detected between France and the UK.

The annual Islamic pilgrimage to Mecca, the Hajj, attracts more than a million pilgrims from many countries worldwide and has been associated with outbreaks of meningococcal disease. The first reported international outbreak of this disease following the Hajj, which was caused by *Neisseria meningitidis* serogroup A, occurred in 1987 (1–3). That epidemic emphasized the potentially high risk of transmission of *N. meningitidis* during the pilgrimage. Before this outbreak, vaccination against *N. meningitidis* serogroup A was required only for pilgrims from sub-Saharan countries to obtain a visa for Saudi Arabia (4). After this outbreak, vaccination became compulsory for all subsequent pilgrims. In 1992, another group A outbreak occurred in Mecca during Umra and Ramadan, but this outbreak was not known to have spread beyond Saudi Arabia (4). Subsequently, the vaccination policy was extended to all Umra visitors.

In March and April 2000, national reference centers (NRC) for meningococci in several European countries detected a sharp rise in isolates of *N. meningitidis* serogroup W135, serotype 2a subtype P1.2,5. This strain, rarely isolated in European countries, belonged to the electrophoretic type 37 (ET-37) clonal complex, which is known to cause hyperendemic disease activity (5). Cases of meningococcal disease caused by serogroup W135 following the Hajj were also reported from Saudi Arabia, the United States, and some countries in North Africa, the Middle East, and Asia (6,7).

In most European countries, according to national policies, prophylaxis was recommended only for close contacts of cases. In France, as soon as the outbreak was recognized (April 8, 2000, week 14), the Ministry of Health recommended a 2-day course of rifampicin for all pilgrims and their household contacts, with the objectives of preventing cases in pilgrims’ households and limiting the spread of the outbreak strain in the population.

We describe the epidemiology of the outbreak in Europe and evaluate the impact of the French intervention.

## Methods

A confirmed case was defined as invasive disease caused by *N. meningitidis* of serogroup W135 2a P1.2,5 or belonging to the ET-37 complex. A probable case was defined as illness in a pilgrim or a pilgrim contact, with either invasive disease due to *N. meningitidis* serogroup W135 of unknown serotype (identified by polymerase chain reaction [PCR] or detection of specific antigens) or with a clinical diagnosis of invasive meningococcal infection without microbiologic confirmation. Cases included were those with dates of hospital admission from March 18, 2000, until July 31, 2000.

Nonpilgrim case-patients were classified as 1) living in the same household as a pilgrim during the 7 days before the date of onset, 2) being in contact with a pilgrim but not living in the same household during the 7 days before the date of onset, or 3) having no identified contact with a pilgrim.

A questionnaire was sent by e-mail in April 2000 to the National Surveillance Centers in Europe, and interviews of cases were conducted by telephone or in person. For each case, information was anonymously requested on demography, ethnicity, clinical symptoms, medical history, housing situation, meningococcal vaccination history, contact with Hajj 2000 pilgrims, and travel to Saudi Arabia. Results of the microbiologic investigation were obtained from NRC. Additionally, the number of visas delivered for the Hajj 2000 was obtained from Saudi embassies, and information was collected in France on the observance of the specific prophylactic measures for pilgrims and their household contacts.

As cases occurred predominantly in France and the UK, only these two countries were considered in further analysis. The case-fatality rate (CFR) observed in cases from France was compared with the CFR of all *N.* *meningitidis* infections reported to the national surveillance system in France during 1995–1999. The same comparison was performed for the UK. The probability of dying from the outbreak strain was estimated for France and the UK by using a logistic regression model. In addition to the outcome (death), variables included in the model were age and having an epidemic case versus a national surveillance case. Consecutively, the age-adjusted relative risks (RR) of dying from the epidemic were estimated from the odd ratios by the log link function.

To evaluate the impact of the French control measures implemented on April 8, we compared the number of cases in France with those in the UK before and after April 8 for a) pilgrim and household contacts, and b) out-of-household contacts and those for whom no contact with a pilgrim was identified. The ratio of cases in France after and before the intervention was compared with the same ratio in the UK. An effective intervention would be expected to result in a measure of impact less than one, and vice versa. Confidence intervals were determined by Poisson regression. The p-values were estimated by Fisher’s exact test.

The descriptive analysis was carried out with Epi Info (Centers for Disease Control and Prevention, version 6.04c). The logistic regression was performed with Stata version 6.0 (Stata Corporation, College Station, TX). Data on cases reported during 1995–1999 were issued from the Public Health Laboratory Service in the UK and from the Institut de Veille Sanitaire in France.

## Results

**Figure 1:**
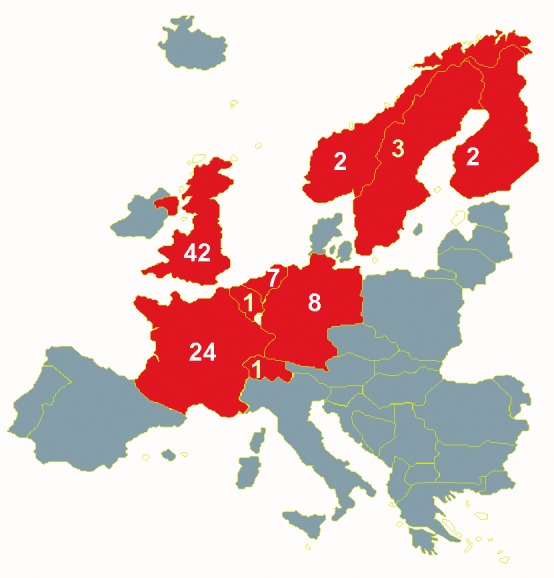
Cases of W135 meningococcal disease reported per country in Europe after Hajj 2000, March 18–July 30, 2000.

From March 18 to July 31, 2000, some 90 cases of serogroup W135 meningococcal disease were ascertained from nine European countries (the UK, France, the Netherlands, Germany, Finland, Sweden, Belgium, Switzerland, and Norway) (Figure 1). Questionnaires were fully completed for 80 cases (89%), and partly completed for 10 cases. Eighty-four isolates (93%) were confirmed as *N.* *meningitidis* serogroup W135 serotype 2a subtype P1.2,5. Six probable cases were diagnosed by soluble antigen detection (one case) or PCR (five cases). Microbiologic examination of bacterial isolates was described elsewhere (5).

**Figure 2:**
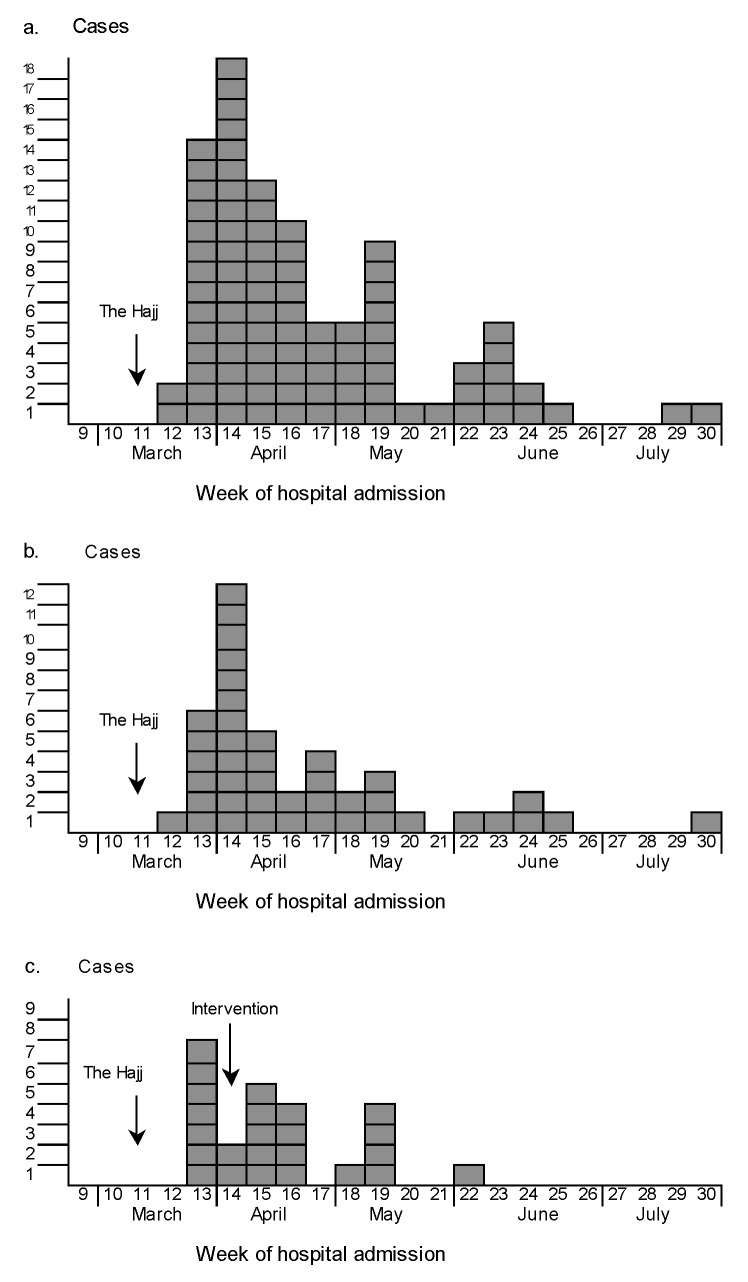
Cases of W135 invasive meningococcal disease by week of hospital admission, March 1–July 2000: a. Europe (90 cases), b. the United Kingdom (42 cases), and c. France (24 cases).

The Hajj 2000 was held March 15–18, 2000 (week 11). The peak of the outbreak was rapidly reached in week 14, two weeks after the first return of pilgrims from Mecca. Since that time, the number of cases regularly decreased (Figure 2a). Of 90 cases, 45 (50%) occurred during the first 4 weeks after the first return of pilgrims. In the UK, the first reported cases occurred within 1 week after the Hajj and increased rapidly to a peak of 12 cases in week 14 (Figure 2b). In France, the outbreak started and peaked in week 13 (Figure 2c). For the other countries, cases occurred sporadically during the 4 months after the Hajj.

Twelve cases (13%) were in pilgrims (all vaccinated with vaccine against meningitis A and C before traveling to Mecca), 31 (34%) in household contacts of a pilgrim, and 21 (23%) in contacts outside the household; for 26 cases (29%), no pilgrim contact was identified.

A total of 19,749 pilgrims from the UK and 19,100 from France participated in the Hajj 2000. Eight cases of meningococcal disease occurred in the UK and four in France, giving incidence rates in the pilgrim population of 41 and 21/100,000, respectively. No cases occurred in pilgrims from other countries, although Germany had 18,000 pilgrims and the Netherlands had 4,500 pilgrims. For Finland, Sweden, Belgium, Switzerland, and Norway, no data were available on visas delivered.

**Figure 3:**
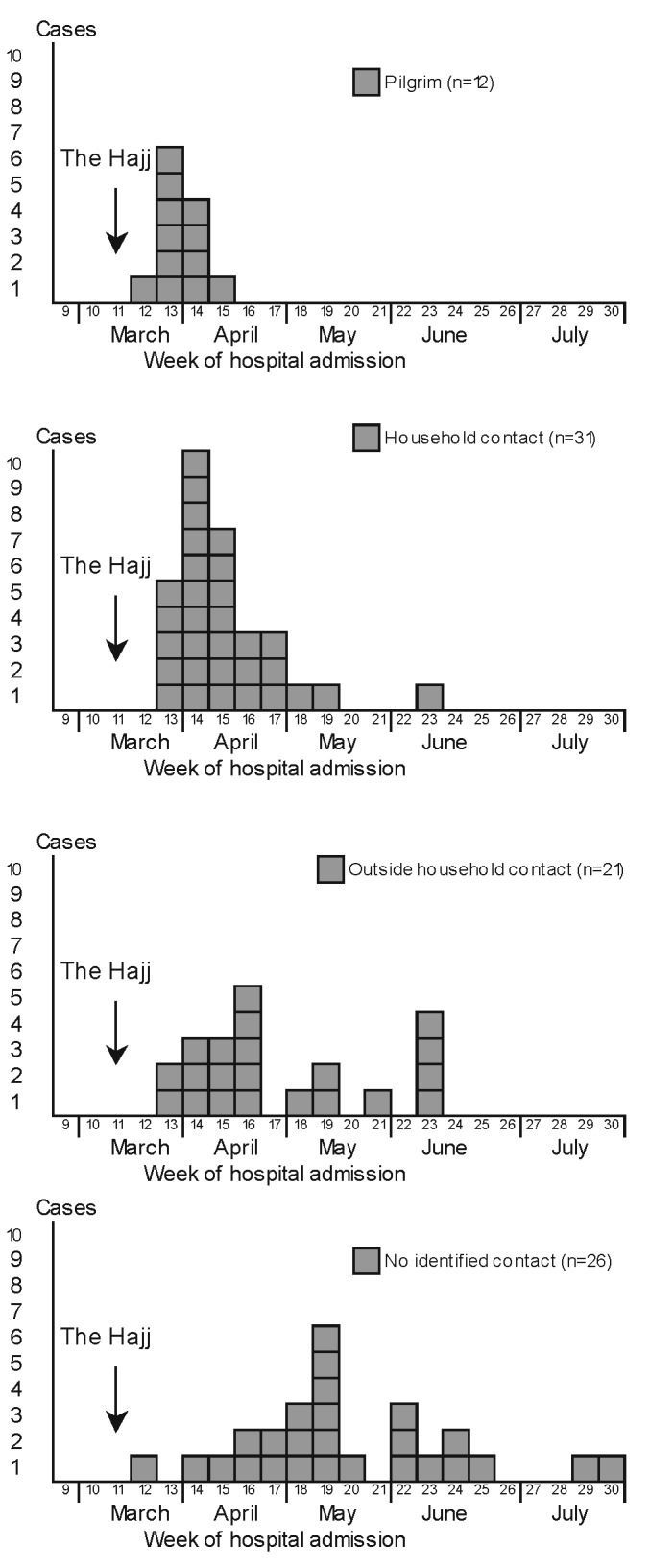
Cases of W135 invasive meningococcal disease, by week of hospital admission and type of contact, Europe, March–July 2000.

Pilgrims were affected first (all cases in pilgrims were reported in the 4 weeks after the Hajj): the peak of cases occurred in week 13, household contacts cases peaked in week 14, out-of-household contact cases peaked in week 16, and cases with unknown or no identified contact with a pilgrim peaked in week 19 (Figure 3).

Forty-seven (54%) of the 87 patients whose sex was known were female. Fifty-one (65%) of the 78 nonpilgrim patients were <5 years of age. The median age of the pilgrim patients was 51 years; for nonpilgrims, it was 2 years. Information on clinical features was available for 69 cases, including meningitis (30 cases), septicemia (23 cases), or both (16 cases). Arthritis (six cases), osteomyelitis (one), and pneumonia (one) were also reported from patients with septicemia. Purpura fulminans was reported in 12 cases. Thirteen patients had underlying long-term diseases, but none had preexisting immunodeficiency. In the UK, 75% of patients were of Indian ethnicity; in France, the majority (74%) were North African.

Fourteen patients died (CFR 15.6%, 14/90) (Table 1). In France, 4 (16.7%) of 24 case-patients died, compared with 152 (10.3%) of 1,481) observed for meningococcal disease due to all serogroups of *N*. *meningitidis* in France from 1995 to 1999. The age-adjusted RR of dying during the outbreak was 1.26, (95% CI [0.52; 3.06], p=0.63). In the UK, 8 (19.0%) of 42 patients died, compared with 862 (8.3%) of 10,448 observed for meningococcal disease due to all serogroups of *N.* *meningitidis* in the UK during1995–1999. The age-adjusted RR was 1.99 (confidence interval 1.02; 2.74, p=0.06). There was no evidence that the RR in France differed from the RR in the UK (p=0.55).

**Table 1:** Cases of W135 meningococcal disease reported from nine European countries following Hajj, 2000

	UK (no., % ; n=42)	France (no., % ; n=24)	All other countries (no., % ; n=24) ^a^	Total (no., % ; n=90)
				
Sex ratio (M/F)	0.6	0.7	1.7	0.9
				
Age distribution				
<1 year	6 (14)	3 (13)	5 (21)	14 (16)
1–4	14 (33)	7 (29)	14 (58)	35 (39)
5–9	6 (14)	2 (8)	0	8 (9)
10–19	0	2 (8)	1 (4)	3 (3)
20–49	7 (17)	3 (13)	1 (4)	10 (11)
50–65	5 (12)	4 (17)	0	10 (11)
>65	4 (10)	3 (13)	1 (4)	8 (9)
Unknown	0	0	2	2
No. of deaths				
<1 year	0	0	0	0
1–4	1	1	2	4
5–9	1	0	0	1
10–19	0	0	0	0
20–49	3	1	0	4
50-64	1	1	0	2
>65	2	1	0	3
				
CFR (overall)	19.0%	16.7%	8.3%	15.6%

^a^The Netherlands, Germany, Finland, Sweden, Belgium, Norway, and Switzerland. CFR, case-fatality rate. UK, United Kingdom.

The median length of stay in Saudi Arabia for pilgrim case-patients was 21 days (range 14–40). The median interval between the beginning of the Hajj and the date of onset of disease was 16 days (range 11–29); the median interval between return from the pilgrimage and the onset of disease was 2 days (range 0–8). Among cases with pilgrim contact, the median interval between return of the pilgrim from Mecca and onset of disease was 8 days for household contacts and 6.5 days for out-of-household contacts (p=0.58). Further characteristics of pilgrim cases and cases by type of contact with a pilgrim are shown in Table 2.

**Table 2:** Characteristics of pilgrim and contact cases in Europe following the Hajj, 2000

	Pilgrims	Household contacts	Outside-of- household contacts	No identified contact
				
No. of cases	12	31	21	26
Mean age (yrs)	50.0	16.0	6.9	25.8
Sex ratio (M/F)	4/8	13/18	13/8	10/13^a^
Median no. of bedrooms in household	3.0	2.5	2.5	2.0
Median no. of rooms in household	6.0	5.0	5.5	4.0
CFR	5/12 (41.7%)	3/31 (9.7%)	1/18 (5.6%)	5/21 (23.8%)

^a^ Sex not known for three cases. CFR, case-fatality rate.

Regarding the assessment of the French recommendations, the comparison between France and the UK of the ratio of cases after and before interventions were put in place was 2.43 for pilgrims and their household contacts. This result of >1 yields no evidence that the French measures had a positive impact in preventing cases in pilgrims and their household contacts (p=0.27) (Table 3). The ratio among out-of-household and no known contacts was 0.56, indicating a possible positive impact of the measures in limiting the spread of the outbreak strain, although the result was not statistically significant (p=0.62).

**Table 3:** Number (and ratio) of cases in United Kingdom (UK) and France before and after a French Ministry of Health recommendation^a^

	UK (n=42)			France (n=24)			Measure of impact (Ratio France / ratio UK)
	No. of cases		Ratio	No. of cases		Ratio	
	Before	After	After/ before	Before	After	After/ before	
Pilgrim cases (a)	8	0		3	1		
Household contacts (b)	9	5		4	4		
(a)+(b)	17	5	0.3	7	5	0.7	2.4 [0.5; 11.1]; p=0.27
Outside-of-household contacts (c)	2	4		2	5		
No identified contact (d)	0	14		0	5		
(c)+(d)	2	18	9	2	10	5	0.6 [0.1; 4.6]; p=0.62
All cases	19	23	1.2	9	15	1.7	1.4 [0.5; 3.8];p=0.61

^a^April 8, 2000. Ratio is divided by the same ratio in the United Kingdom and its 95% confidence interval.

## Discussion

This report is the first description of a European meningitis outbreak involving nine countries. An international coordination group was set up within 2 weeks after the outbreak was recognized in Europe, followed by an early warning alert to all the European countries. This facilitated information sharing, standardization of the case definition, and implementation of a standardized questionnaire for the investigation. The willingness of participant countries led to a satisfactory completion rate for reports, allowing a precise description of the outbreak throughout Europe. However, case ascertainment may have varied in different countries since some countries identified cases through microbiologic findings only and some through clinical as well as microbiologic findings.

In 1987, an outbreak of meningococcal disease linked to the Hajj was described in several European countries, but the description was limited to pilgrim cases and a few secondary cases (1–3). In the Hajj 2000 outbreak, the added value of molecular biological investigation, together with the epidemiologic investigation, allowed us to describe a W135 clonal outbreak and the diffusion of this strain from pilgrims to the general population (5). In Sweden, *N.* *meningitidis* W135 of the same serotype and subtype has been documented since 1979, but pulsed-field gel electrophoresis and the sulfadiazine resistance of the W135 isolates indicate that the outbreak was probably due to a new strain of W135 meningococci (8).

After its description in 1968 and during the 1970s, *N. meningitidis* W135 was considered a minor serogroup, of little clinical importance (9). Only in the early 1980s was this organism described as a fully pathogenic strain, as an important new cause of disease in Europe and the United States and as an emerging cause of meningococcal disease in Africa (10,11). During the 1990s, *N. meningitidis* W135 represented 2.6% to 4% of all reported *N. meningitidis* in the UK, France, and the United States (12–14). The first two cases of meningococcal disease in pilgrims due to W135 associated with the Hajj were described in 1993 in Saudi Arabia in an Indonesian and an American pilgrim (15).

From 1998 to March 2000, fewer than two cases of the W135 2a:P1-5,2 strain were reported yearly in England and Wales (Kaczmarski EB, pers. comm.) and two cases per year in France (Taha MK, pers. comm.). The outbreak strain belonged to the ET-37 complex, which is mainly composed of serogroup C (16). The ET-37 complex has caused hyperendemic disease activity and outbreaks worldwide. It causes disease in clusters and has a higher transmissibility than other strains (5,17,18).

Although the CFRs in cases from France and the UK were high, the age-adjusted RRs of dying during the outbreak were not significantly higher than those observed in the routine surveillance of meningococcal disease due to all serogroup of *N. meningitidis* in these two countries. Thus, the outbreak strain appears to be of similar virulence to *N. meningitidis* serogroups that normally cause meningococcal disease in the UK and France. CFRs have been shown to be linked with age and to increase among very young and older people (19). The initially large number of cases in older people at the beginning of the outbreak might explain this finding.

Methods used to evaluate the impact of the specific control measures implemented in France intended to take into account differences in meningococcal disease incidence rates between the two countries and potential differences in pilgrims’ initial carriage of the outbreak strain at their return from Mecca. Since the number of cases in each group (pilgrims, household, out of household, and no identified contact with a pilgrim) was low, the power of the test did not allow identification of the difference of impact between them. Defining other groups could not have allowed conclusions to be drawn, since the number would have also been low. Information collected from the only manufacturer of rifampicin in France indicated that the total number of doses distributed to pharmacies represented only half the doses needed to treat the target group (approximately 100,000 persons living in pilgrims’ households), indicating that compliance with the recommendations was low. In addition, all those who were provided treatment may not have taken it effectively, although compliance would not be expected to be a major problem with only 2 days of medication. As of the end of March 2001 in France, no cases were reported in persons who had taken rifampicin and no strain of *N. meningitidis* W135 resistant to rifampicin had been isolated at the NRC. For the prevention of cases among pilgrims and household contacts, the <1 ratio between France and the UK indicated that the measure had no impact in preventing cases in pilgrims and pilgrims’ household in France. This might be due to the delay of 2 weeks between the first return of pilgrims and the release of the measure, an interval during which transmission of the pathogenic strain occurred inside the pilgrims’ households**.**

The absence of significant impact of the measure to limit the diffusion of the pathogenic strain to the out-of-household contacts and persons with no contacts identified may be explained either by the very small number of cases considered or by potential misclassification. Cases in the general population may also have been underestimated in comparison with the likely high case ascertainment in pilgrims. However, such underestimates are unlikely since virtually all invasive strains of *N. meningitidis* are sent to the reference laboratory in France and the UK. However, data for cases of W135, 2a P1.2,5 obtained from national reference laboratories in France and the UK for September 2000–February 2001 indicated that 13 cases were reported in the UK and 9 in France, suggesting that there was no long-lasting effect of the measure and that immunity to the strain was probably increasing in the population (20). In the UK, carriage studies showed that this strain was still circulating within the Muslim community (Stuart JM, pers. comm.). The results of the measures implemented in France do not allow us to draw conclusions for use of mass prophylaxis in the future, mainly because of the small number of cases in our study.

Following this outbreak, France and the UK, among other countries, recommended quadrivalent vaccine for travelers to the Hajj 2001 (21,22; pers. comm., Secrétariat du Conseil Supérieur d'Hygiène Publique de France). Subsequent quadrivalent vaccine coverage was estimated to be 47% and 65% in the UK and in France, respectively. Another outbreak of meningococcal disease caused by *N. meningitidis* W135 2a P1.2,5 occurred in Hajj pilgrims and their contacts in 2001; most cases were from Saudi Arabia and the UK. During the period March 28–June 29, 2001, 10 cases of meningococcal disease due to W135 2a P1.2,5 were reported to the NRC in France (0 deaths) and 25 in the UK (8 deaths) (23–25). Since May 2001, quadrivalent vaccine is now a requirement for all pilgrims to future Hajj pilgrimages (26).
